# Supplementary material for: Porous single-crystalline titanium dioxide at 2 cm scale delivering enhanced photoelectrochemical performance
Source: Nat Commun. 2019 Aug 9;10:3618. doi: 10.1038/s41467-019-11623-w (PMC6689047; doi:10.1038/s41467-019-11623-w)
Supplement: Supplementary file 1 — Supplementary Information [file 41467_2019_11623_MOESM1_ESM.pdf]

Supplementary Information

Porous single-crystalline titanium dioxide at 2 cm scale delivering enhanced photoelectrochemical performance

Cheng et al.

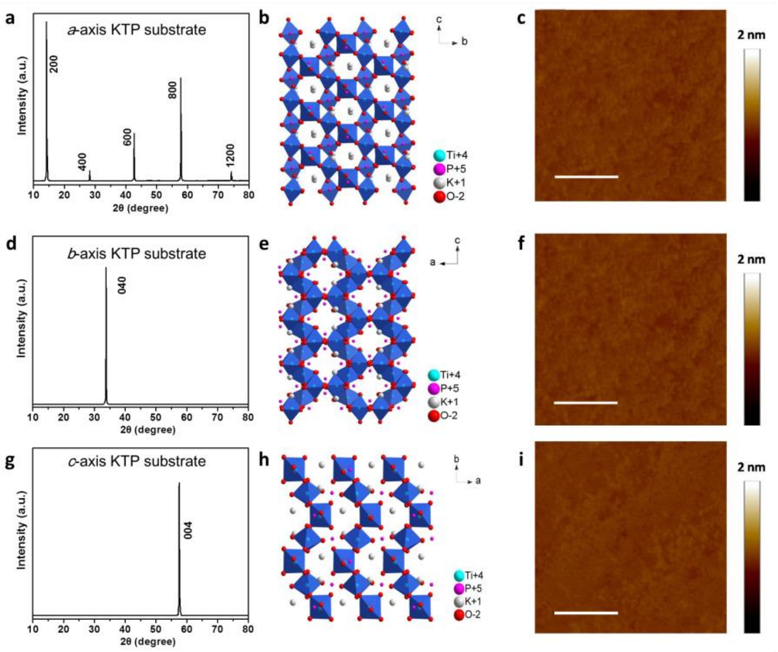

Supplementary Fig. 1. Crystal structure and surface microstructure of KTP crystals. XRD patterns (**a**, **d** and **g**), crystal structure (**b**, **e** and **h**) and mean surface roughness (**c**, **f** and **i**) along the *a*-axis, *b*-axis and *c*-axis of KTP substrate used to grow P-SC TiO_2 crystals, respectively. The scale bar is 500 nm and the roughness is ~0.1 nm in **c**, **f** and **i**.

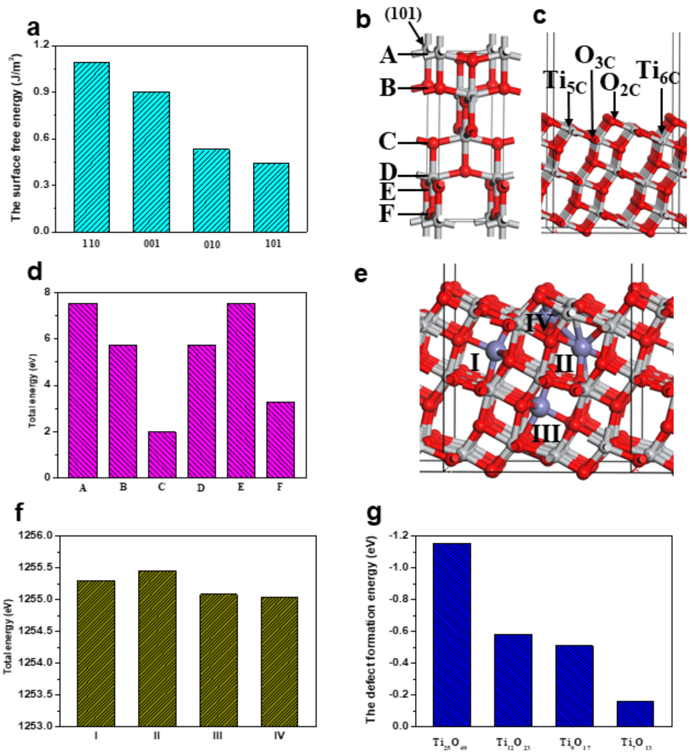

Supplementary Fig. 2. The energy and structures of Ti_nO_{2n-1} system. (**a**) The surface free energy of different crystal facets; (**b-c**) The optimized structure of anatase TiO_2 and the TiO_2 (101) surface, O atoms in red, Ti atoms in silvery; (**d**) The total energy of the TiO_2 (101) surface with different atomic terminations; (**e**) The structure of anatase TiO_2 (101) surface with one interstitial Ti atom marked in purple to simulate $Ti_{25}O_{49}$ crystal; (**f**) The total energy of the TiO_2 (101) with different position of interstitial Ti atom, I-IV corresponds to the four interstitial positions in lattice; (**g**) The defect formation energy of Ti_nO_{2n-1} system.

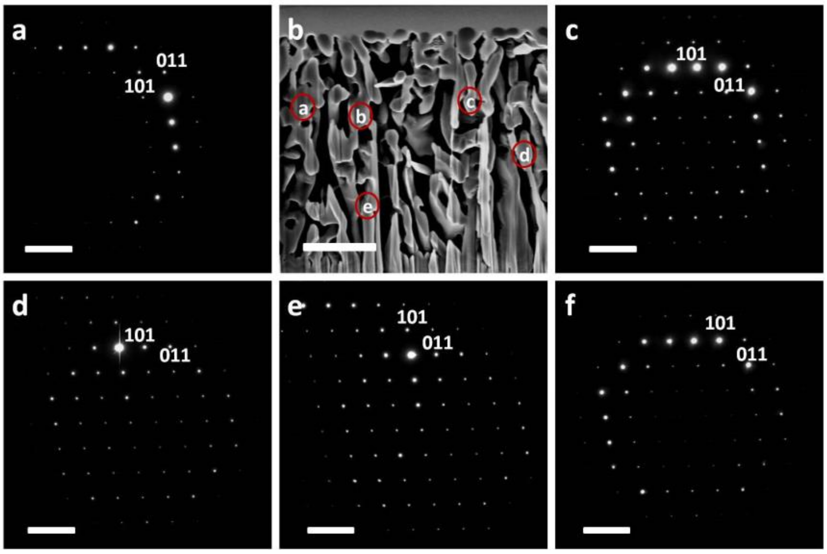

Supplementary Fig. 3. Microstructure of the P-SC $Ti_{38}O_{75}$ grown along the *b*-axis of KTP substrate. Cross-sectional TEM characterization (**b**) and corresponding five SEAD patterns (**a**, **c**, **d**, **e** and **f**) at different locations on the skeleton of P-SC $Ti_{38}O_{75}$ grown along the *b*-axis of KTP crystal substrate. The scale bar is 5 1/nm in **a** and **c**-**f**. The scale bar is 1 μm in **b**.

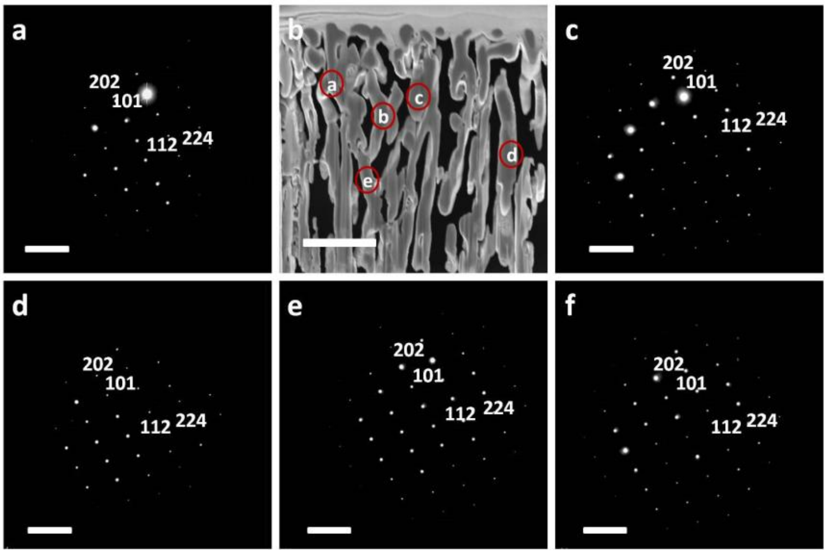

Supplementary Fig. 4. Microstructure of the P-SC $Ti_{38}O_{75}$ grown along the *c*-axis of KTP substrate. Cross-sectional TEM characterization (**b**) and corresponding five SEAD patterns (**a**, **c**, **d**, **e** and **f**) at different locations of the skeleton of P-SC $Ti_{38}O_{75}$ grown along the *c*-axis of KTP crystal substrate. The scale bar is 5 1/nm in **a** and **c**-**f**. The scale bar is 1 μm in **b**.

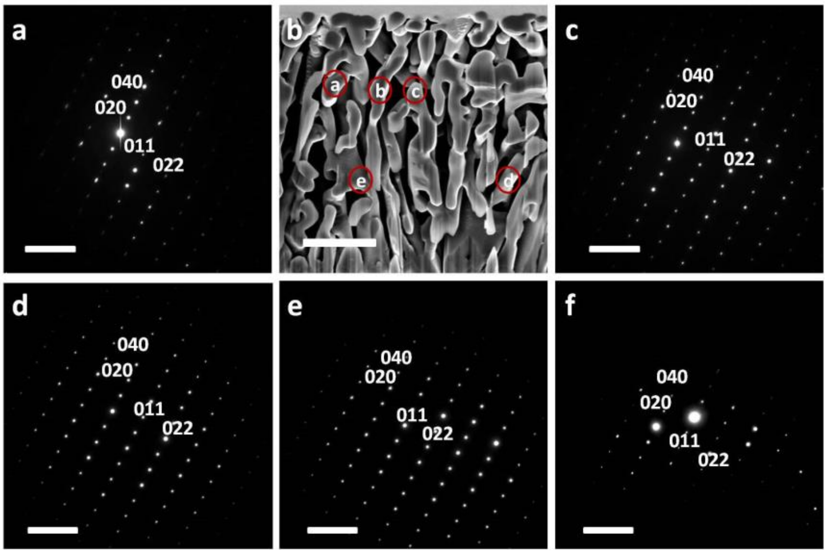

Supplementary Fig. 5. Microstructure of the P-SC $Ti_{38}O_{75}$ grown along the *a*-axis of KTP substrate. Cross-sectional TEM characterization (**b**) and corresponding five SEAD patterns (**a** and **c**-**f**) at different locations at different locations of the skeleton of P-SC Ti_9O_{17} grown along the *a*-axis of KTP crystal substrate. The scale bar is 5 1/nm in **a** and **c**-**f**. The scale bar is 1 μm in **b**.

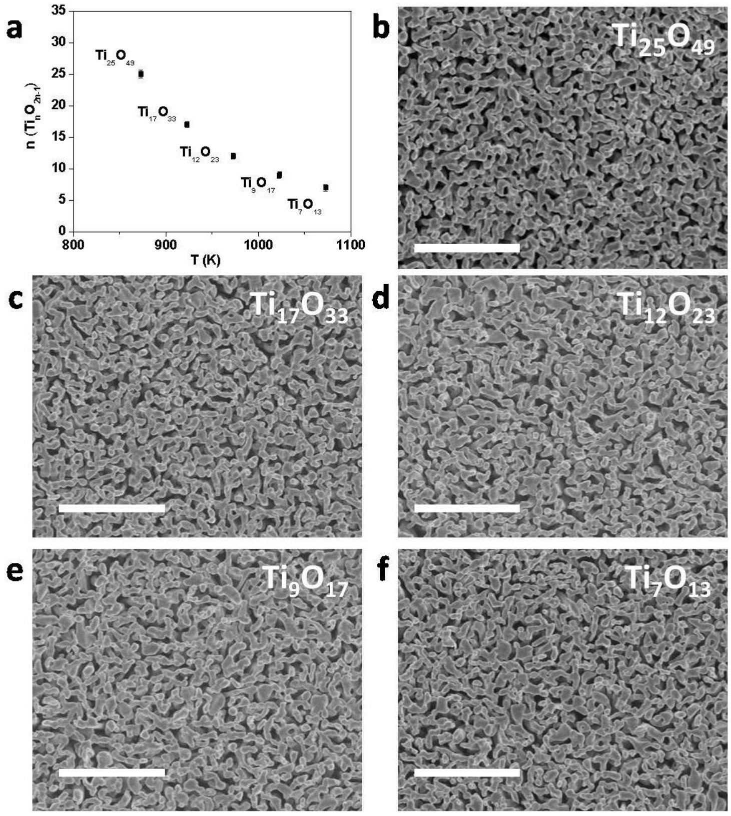

Supplementary Fig. 6. Chemical composition and morphology of P-SC Ti_nO_{2n-1} crystals. a, The n values of P-SC Ti_nO_{2n-1} crystals are confirmed by direct detection of CO generation when reducing Ti_nO_{2n-1} crystals with graphite in vacuum system. SEM images of P-SC Ti_nO_{2n-1} crystals grown along the *a*-axis of KTP substrate at various growth temperatures. **b**, 600 ^oC; **c**, 650 ^oC; **d,** 700 ^oC; **e,** 750 ^oC and **f** 800 ^oC. The scale bar is 2 μm in **b**-**f**.

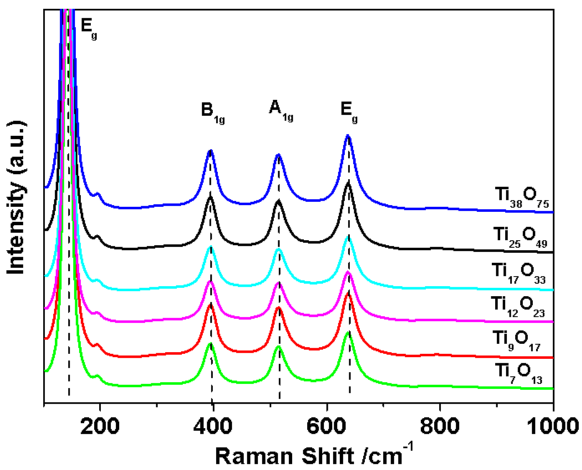

Supplementary Fig. 7. Raman spectra of P-SC Ti_nO_{2n-1} crystals with excitation lines at 532 nm. The typical peaks well fit the anatase TiO_2 while slight Raman shift is observed with different chemical compositions.

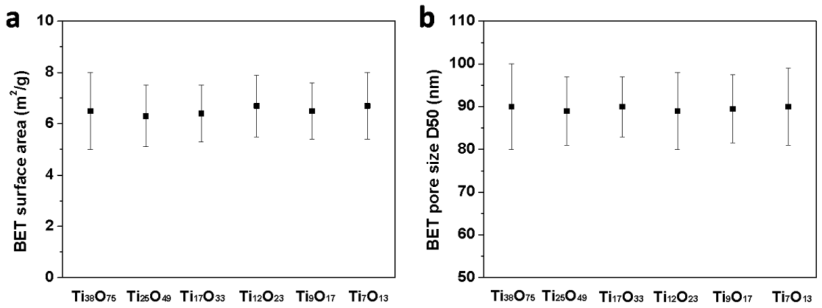

Supplementary Fig. 8. Surface area and pore size of the P-SC Ti_nO_{2n-1} crystals. (a). BET surface area of P-SC Ti_nO_{2n-1} crystals. (b). BET mean pore size of P-SC Ti_nO_{2n-1} crystals.

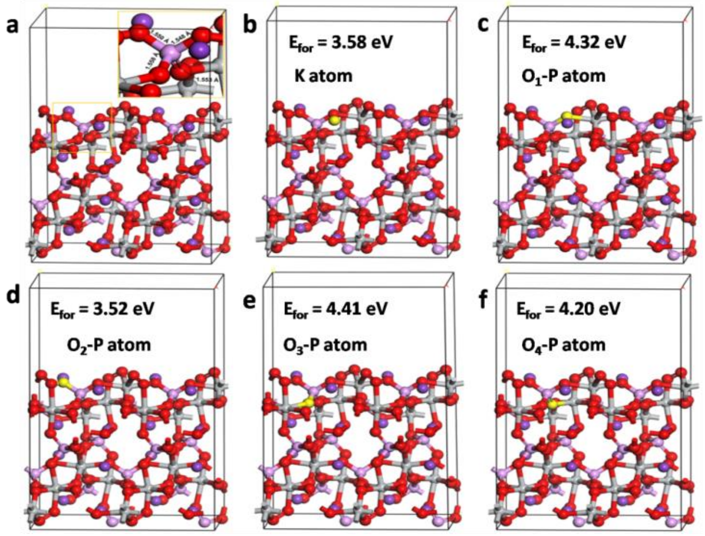

Supplementary Fig. 9. The different defect configurations of the KTP (100) surface. (**a**) Perfect KTP (100) surface, (**b**) K atom defect and (**c-f**) different O atom defects on KTP (100) surface. The defect formation energies (E_{for}) are marked in the top.

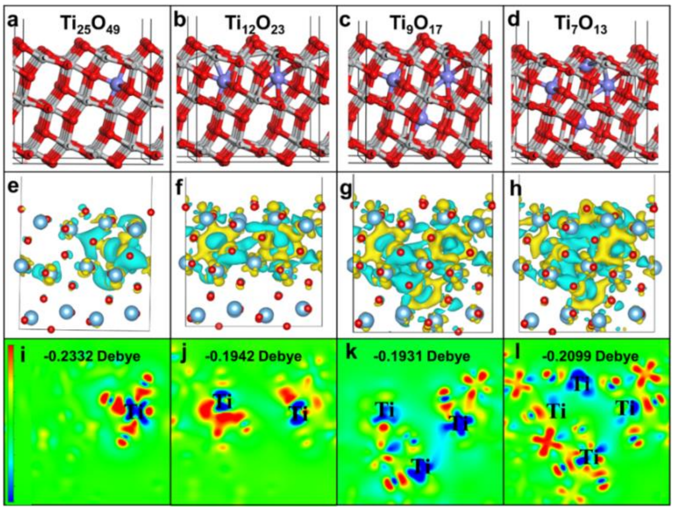

Supplementary Fig. 10. The structures and charge density difference of Ti_nO_{2n-1} (101) system. (**a**-**d**) The optimized structures of Ti_nO_{2n-1} (101); O atoms in red, Ti atoms in silvery and the interstitial Ti atoms marked in purple for clear; (**e**-**h**) the charge density difference of Ti_nO_{2n-1} (101); The accumulation and loss of charge are represented by yellow and blue regions, respectively; (**i**-**l**) the electronic charge density difference of Ti_nO_{2n-1} (101); the dipole moment of Ti_nO_{2n-1} (101) along the z direct listed on the plots.

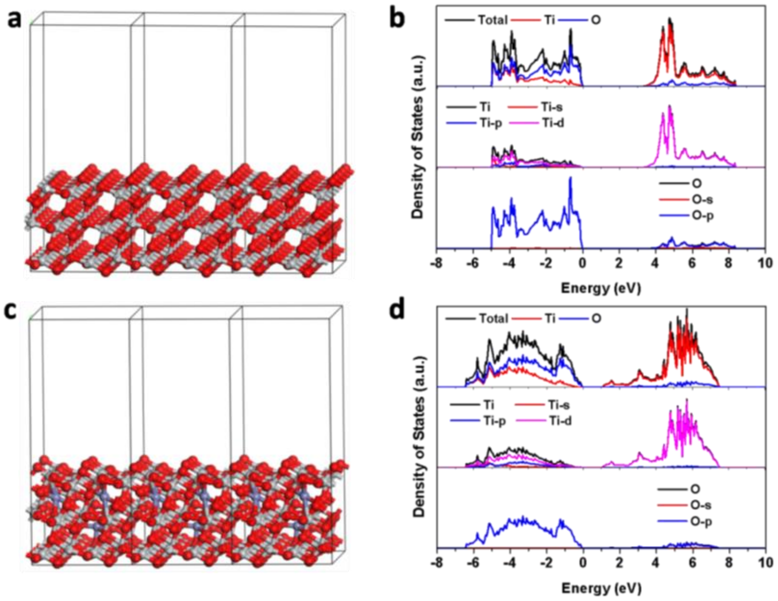

Supplementary Fig. 11. Electronic structures with and without Ti interstitials. The optimized configurations of TiO_2 (101) - (1×4) surface unit cell and the projected density of states (PDOS). (**a**, **b**) for the pure TiO_2; (**c**, **d**) for involving Ti atoms as interstitial in TiO_2.

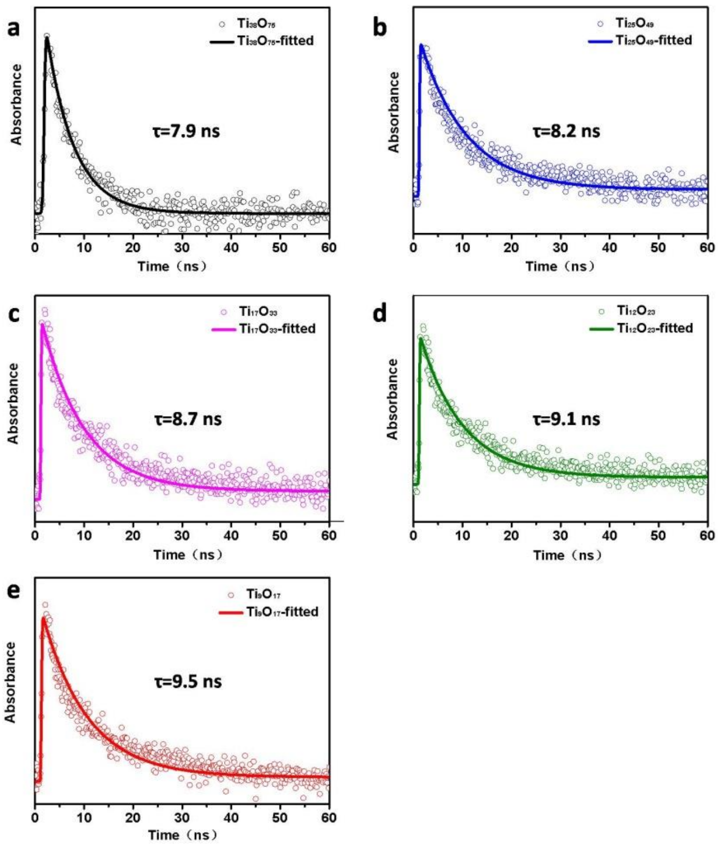

Supplementary Fig. 12. Decay profiles of transient absorption of P-SC Ti_nO_{2n-1}. (**a**) $Ti_{38}O_{75}$, (**b**) $Ti_{25}O_{49}$, (**c**) $Ti_{17}O_{33}$, (**d**) $Ti_{12}O_{23}$ and (**e**) Ti_9O_{17}.

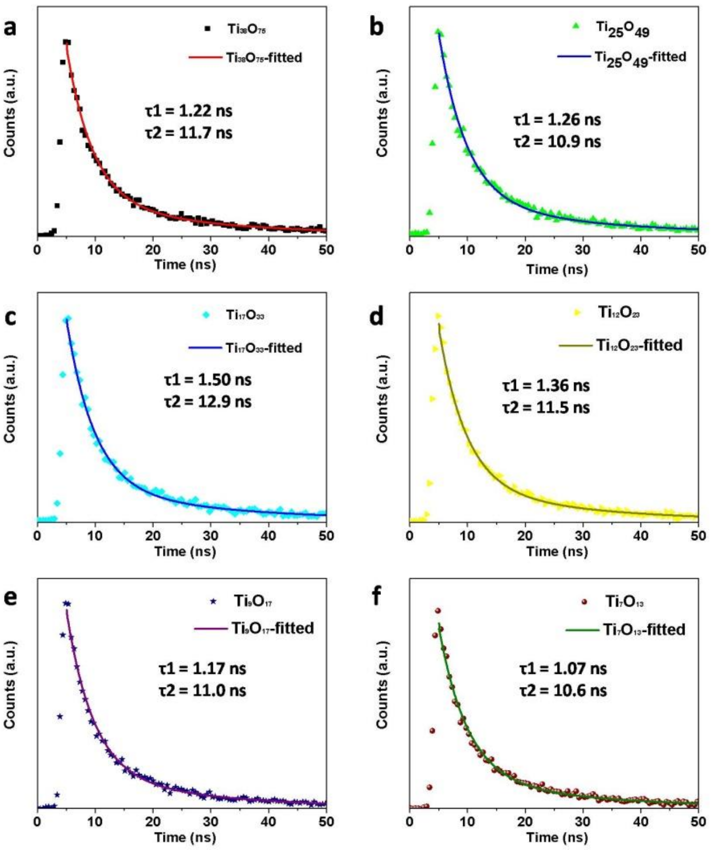

Supplementary Fig. 13. The fluorescence decay curves of P-SC Ti_nO_{2n-1}. (**a**) $Ti_{38}O_{75}$, (**b**) $Ti_{25}O_{49}$, (**c**) $Ti_{17}O_{33}$, (**d**) $Ti_{12}O_{23}$, (**e**) Ti_9O_{17} and (f) Ti_7O_{13}.

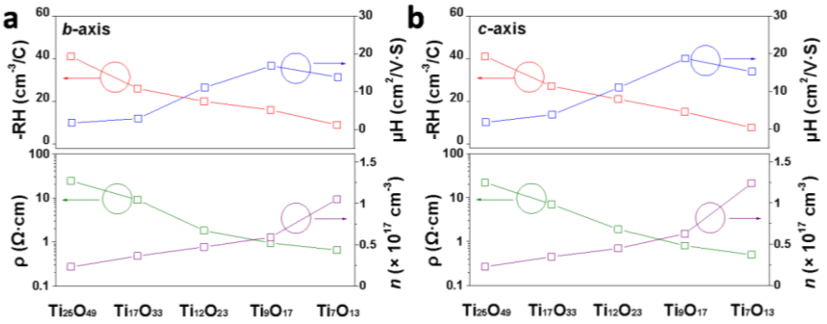

Supplementary Fig. 14. **Transport properties of P-SC Ti_nO_{2n-1} crystals.** (**a**) The resistivity, carrier density, Hall coefficient and Hall mobility of Ti_nO_{2n-1} single crystals growth along the *b*-axis of KTP substrates. (**b**) The resistivity, carrier density, Hall coefficient and Hall mobility of P-SC Ti_nO_{2n-1} (n = 7-25) single crystals growth along the *c*-axis of KTP substrates.

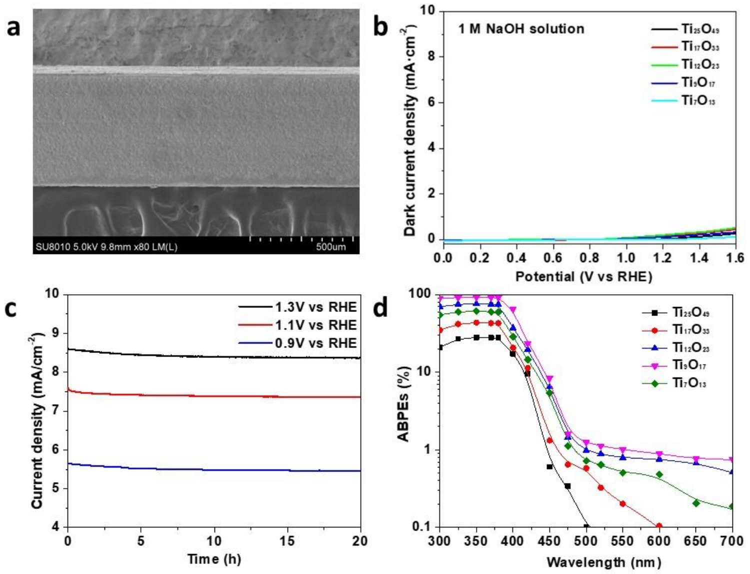

Supplementary Fig. 15. Microstructure and photoelectrochemical performance. (**a**) The cross-sectional view of the free-standing P-SC Ti_7O_{19} single crystal electrode. (**b**) The dark current densities of the P-SC Ti_nO_{2n-1} (n = 7-25) photoanodes in 1M NaOH electrolyte solution. (**c**) Durability test of the P-SC Ti_9O_{17} at different bias. (**d**) The ABPEs curves of the P-SC Ti_nO_{2n-1} (n = 7-25) at 1.23 V photoanodes in 1M NaOH electrolyte solution. The scale bar is 0.5 mm in **a**.

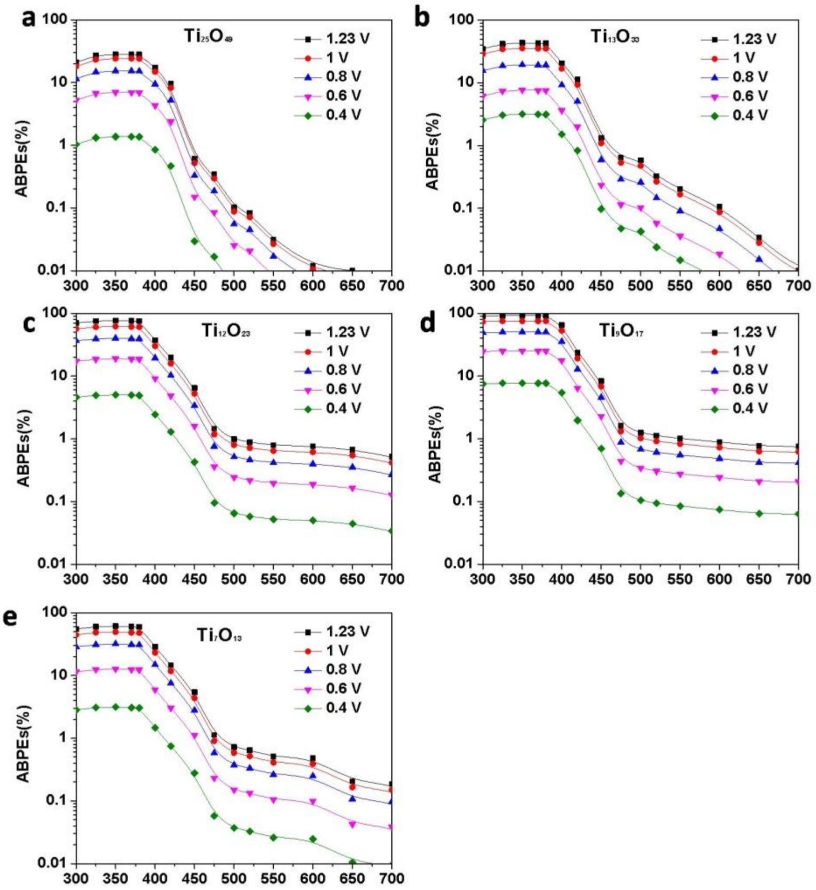

Supplementary Fig. 16. The ABPEs curves of the P-SC Ti_nO_{2n-1} (n = 7-25) photoanodes in 1M NaOH. (**a**) $Ti_{25}O_{39}$, (**b**) $Ti_{17}O_{33}$, (**c**) $Ti_{12}O_{23}$, (**d**) Ti_9O_{17} and (**e**) Ti_7O_{13}. The ABPEs are measurement at different bias

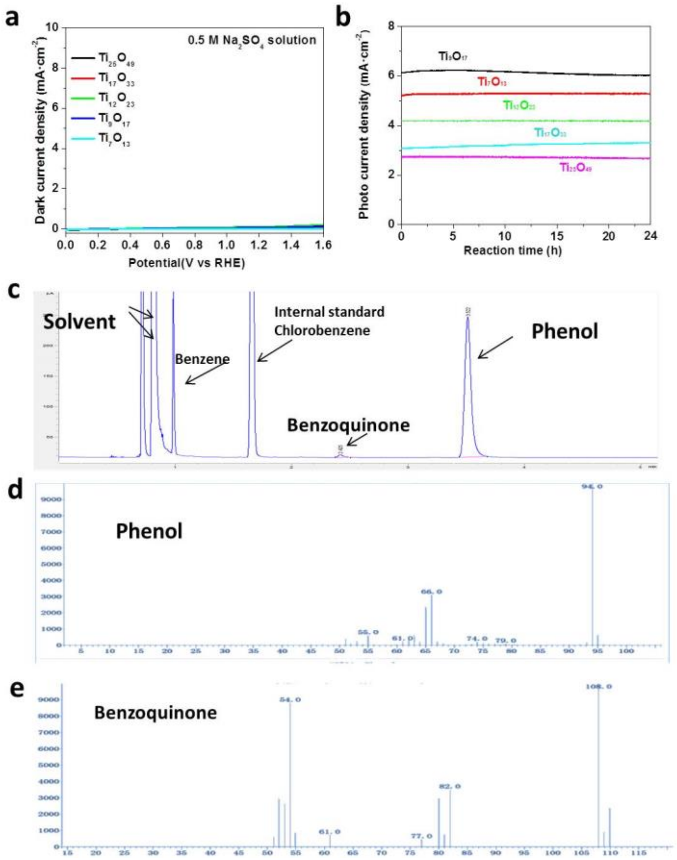

Supplementary Fig. 17. Photoelectrochemical oxidation of benzene to phenol. (**a**) The dark current curves of the P-SC Ti_nO_{2n-1} (n = 7-25) photoanodes in 0.5M Na_2SO_4 electrolyte solution. (**b**) Durability test of the P-SC Ti_nO_{2n-1} (n = 7-25) photoanodes in 0.5M Na_2SO_4 electrolyte solution. (**c**) Gas chromatograph spectrum for products of photoelectrochemical oxidation of benzene on P-SC Ti_9O_{17} photoanode. (**d**) and (**e**) Mass spectroscopy results of the main product (Phenol) and by-product (Benzoquinone).

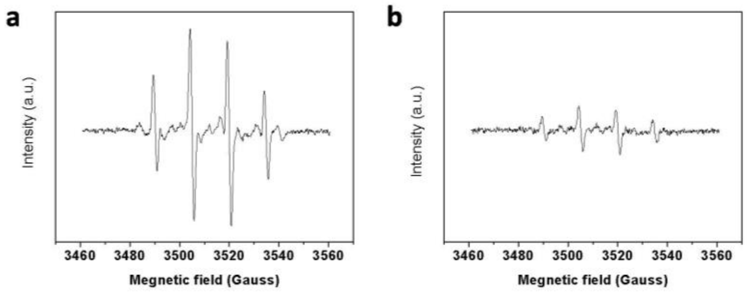

Supplementary Fig. 18. Electron spin resonance (ESR) signal of •OH radical. DMPO spin-trapping ESR spectra for the •OH radical in the presence of porous single-crystalline Ti_7O_{19} under different light intensities. (**a**) 10 ×AM 1.5G irradiation and (**b**) 1 ×AM 1.5G irradiation.
